# Effect of COVID-19 on dental service delivery in Fiji: Perspective of Dental Officers (Dos) and Dental Managers (DMs)

**DOI:** 10.1371/journal.pone.0287886

**Published:** 2023-06-27

**Authors:** Kartika Kajal, Masoud Mohammadnezhad

**Affiliations:** 1 School of Dentistry and Oral Health, Fiji National University, Suva, Fiji; 2 School of Nursing and Healthcare Leadership, University of Bradford, Bradford, United Kingdom; 3 Faculty of Public Health, Department of Health Education and Behavioral Sciences, Mahidol University, Nakhon Pathom, Thailand; University of Sharjah, UNITED ARAB EMIRATES

## Abstract

**Background:**

Corona Virus Disease 2019 (COVID-19) was declared a global pandemic by the World Health Organization (WHO) has had significant impact on dentistry in Fiji. Due to lack of previous study, this study aims to explore the perspective of Dental Officers (DOs) and Dental Managers (DMs) on the effects of COVID-19 on dental service delivery in Fiji Islands.

**Methods:**

This qualitative study was conducted amongst 30 DOs and 17 DMs between 9th August to 12th September, 2021. It was conducted in the government dental clinics, private dental clinics and the School of Dentistry and Oral Health clinic (SDOH), in the Central Division, Fiji. The study settings were randomly selected. Purposive sampling method was used for the selection of participants who met the study criteria. Semi-structure open ended questionnaires were used for data collection through in-depth interviews via zoom. Manual thematic analysis of the data was conducted to derive themes and codes.

**Results:**

The participants interviewed for the study included more female DOs (66.7%) and male DMs (58.8%). Seven themes emerged from data analysis: range of services delivered, appointment versus walk-in patients for aerosol generating procedures (AGPs), impact of pandemic on clinic opening hours, impact of COVID-19 on patient numbers, quality of services delivered, resources and infrastructure, perceptions about the burden of disease.

**Conclusion:**

COVID-19 has significantly affected dental service delivery. Mostly emergency dental services were delivered. AGPs were delivered on appointment basis. Most participants stated the quality of services had improved. Participants stated that they were not given adequate resources and the infrastructure was not up to standard to provide dental services during the pandemic. The dental disease burden had increased during the pandemic as per the participants. Future research can be conducted amongst other dental professionals in other divisions of the country.

## Introduction

Corona Virus Disease 2019 (COVID-19) is a respiratory disease which emerged from a novel corona-virus [1,2]. It came into limelight in December, 2019 and was declared a global pandemic by the World Health Organization (WHO) [3,4]. Since its emergence, this pandemic has spread worldwide has become the number one health challenge [5,6]. The virus can potentially spread through droplets or by usual contact [7]. The most common symptoms of COVID-19 are fever, cough, sore throat, fatigue, myalgia, headache, shortness of breath and in some cases diarrhea [2,6–8].

COVID-19 has created significant impact on dentists and oral health professionals [9–12]. The scientific literature has drawn attention to factors such as dentists close contact with the patients. This causes constant exposure to body fluids such as blood and saliva as well as the spread of aerosols during dental procedures [12]. Due to the nature of the dental settings, the profession of dentistry has a high risk to transmission of the virus from a dental professional to another or to patients [3,13–16]. The US Centers for Disease Control and Prevention (CDC) has stated that the droplets and aerosols generated during the dental procedures is regarded as high risk [9,17]. The pandemic has led to a reduction in dental services all over the world [4]. Considerable adjustments to the type of services and infection control protocols were required [18] as the risk of infection cannot be controlled though standard Personal Protective Equipment (PPE) in daily dental practice [19].

COVID-19 has significantly affected Fiji and its people. Fiji experienced its first wave in March 2020 while the second wave began in April 2021. As of 29^th^ November 2021, Fiji recorded a total of 52,506 confirmed cases of COVID-19. Out of these, 51,037 have recovered [20]. Fiji has recorded 696 deaths in total since the first outbreak in March 2020. Around the same period, there were about 252 million confirmed cases and 5 million deaths due to COVID-19 globally [21].

Oral health services are provided by both the public and private sector in Fiji. The cost of dental treatment in the public system is quite minimal. Oral health services are provided under four main areas: emergency services; preservative and conservative dentistry; oral health education; and specialized clinical dentistry, which includes oral surgery, prosthodontics, orthodontics, oral medicine, and pediatric dentistry. Community public dental services include oral health community outreach programs and the school oral health programs. Part of the community public health program include oral health education and limited treatment to primary school children using mobile dental clinics. Prevention programs based at the community level, such as fissure sealant and water fluoridation, have been in place for several years [22]. Apart from these, most of the services provided at the school of Dentistry and Oral Health clinics as part of undergraduate training is free of charge for the public. There have been dramatic changes made with regards to delivery of dental services during the pandemic.

The pandemic had caused unpreceded changes to the dental settings, dentists and dental patients. However, no research has yet been conducted in Fiji to report these findings. Hence, this study aims to explore the perspective of Dental Officers (DOs) and Dental Managers (DMs) on the effects of COVID-19 on dental service delivery in Fiji Islands.

## Methodology

### Study design and setting

A qualitative study was conducted among DOs and DMs through in-depth interviews in Central Division, Suva, Fiji between 9th August to 12th September, 2021. A qualitative study helps to collect genuine ideas and provides valuable insights on relevant issues and experiences. It helps to explore and understand social and behavioral issues as well [23–25].

The study was conducted in the government dental clinics, private dental clinics and the School of Dentistry and Oral Health clinic (SDOH). There are approximately 10 government dental clinics including the main Colonial War Memorial Hospital (CWMH) dental clinic, 26 private dental clinics and 1 SDOH in the Central Division, Suva, Fiji. Nine private dental clinics and eight government dental clinics were selected based on random sampling. As for school, there is only one dental school in Fiji which was selected for the study.

### Study sample

The study population comprised of all the dental staff of the dental clinics in Suva, Fiji, serving the public during the pandemic. The study sample on the other hand was selected based on the inclusion and exclusion criteria. The inclusion criteria for DOs included; DOs (Dentists) and dental interns of any ethnicity and gender with at least six months working experience. The exclusion criteria included any other dental practitioners, DOs from other dental clinics and those DOs who were not willing to participate in the study. The inclusion criteria for DMs included; Sub-divisional Dental Officer (SDDO), Senior Dental officers (SDO) and Principal Dental officers (PDO) of the selected government and private dental clinics, DMs of any ethnicity and gender with at least six months working experience. The exclusion criteria included DMs of other clinics apart from the selected dental clinic and DMs who do not provide consent or not willing to participate in the study.

Purposive sampling method was used for the selection of DOs and DMs. Thirty DOs out of approximately 40 were selected for the study based on the inclusion and exclusion criteria; six from private dental clinics, 18 from government dental clinics and six from SDOH. All 30 DOs had undergone an in-depth interview via zoom until data saturation was reached. Seventeen DMs out of approximately 35 were selected based on the exclusion and inclusion criteria who had undergone an in-depth interview via zoom until data saturation was reached; nine from private dental clinics and eight from government dental clinics.

### Data collection tool

A self-developed semi-structure open ended questionnaire was used for data collection through in-depth interviews for DOs and DMs. Semi-structured in-depth interviews is one of the common methods used in qualitative studies to collect data in health service research [26]. It involves dialogue between the researcher and participant and guided by a flexible interview protocol [26]. This method of data collection enables the researcher to collect open- ended data, share feelings and beliefs about a particular topic, explore thoughts of participants and also helps to explore more deeply into personal and sensitive issues as well [26]. The questionnaires had two sections respectively. The first section recorded the demographic information for the DOs and DMs; unique identification number, age, gender, ethnicity, the participants highest level of qualification, and the number of years of practice of the DOs and DMs. The second section included six open ended questions to gauge the dentist’s perception about impact of COVID-19 on dentistry.

### Study procedure

The dental managers of the respective clinics received the flyers two weeks prior to commencing the data collection via email. The flyer contained brief information regarding the study. The DMs were requested to inform their staff regarding the study as well. The interested DMs and DOs emailed and called the principal investigator directly for participation. An interview time was selected by the principal investigator based on the availability of the participants. Each participant was given a participant information sheet. Following this, those participants who agreed to take part in the research were sent a consent form via email. The consent forms were collected and kept by the principal investigator safely. The in depth interviews were conducted for each participant by the principal investigator via zoom ranging from 30 to 35 minutes. An abductive approach of interview was undertaken, whereby the results from the first interview guided the subsequent ones [27]. Voice recording was done as a means of back up and written notes were also taken during each interview. Participants were informed that the interviews shall be recorded and consents were obtained accordingly.

### Data management and analysis

Immediately after each interview, it was transcribed manually by the principal investigator into Microsoft word. After the initial interview was transcribed, the principal investigator read the transcript repeatedly to identify any potential errors which were considered and improved in subsequent interviews [27]. The principal investigator read the transcript multiple times also to become familiar with the content and identify common and significant elements to create codes. Codes are shorthand labels to describe the contents of the interviewee [28]. These codes were grouped (subthemes) to identify common patterns to create broader themes [28] which were reviewed and confirmed by the principal supervisor. Data was interpreted in the context it was obtained to see if the interviewer had any influence on the participants answers and if the answers were requested or not. Data was entered after each interview until data saturation was reached [27].

### Study rigor

It is important to ensure methodological rigour when conducting qualitative studies [29]. Four-Dimensions Criteria (FDC) to establish trustworthiness which has been applied to this study as well; credibility, dependability, confirmability and transferability [29]. Credibility was ensured by engaging with participants, distribution of flyers, verbal explanation regarding the study, and the in-depth interviews ranged from 30–60 minutes. Dependability was maintained by having a thorough literature search, data coding was done and transcripts were re-read to identify errors, and raw data were kept by the principal investigator. Confirmability was maintained in the following ways: thorough methodology and investigator and data source triangulation were ensured. To ensure transferability, random and purposive sampling methods were used, and data was collected until data saturation was reached.

### Ethical considerations

Ethics approval were taken from the College Health Research and Ethics Committee (CHREC) of Fiji National University (FNU), and Fiji National Health Research and Ethics Review Committee (FNHRERC) and facility approval from various private dental clinics selected for the study. Written consent form was obtained from them before collecting data. Participant confidentiality was maintained at all times using unique identification numbers (codes) instead of using their names. The participants were informed that their participation in the study is voluntary and they could leave the study at any stage. The transcribed scripts and recorded interviews were only accessible to the principal investigator and was kept in a computer which was password protected.

## Results

### Characteristics of DOs and DMs

There were more female DOs (66.7%) compared to male (33.3%). Majority participants were from the age range of 20–30 years (50%) while only 3.3% was more than 60 years of age. 80% participants were Fijians of Indian Descent (FID), 10% were I-taukei (IT) while 10% belonged to other ethnicities. Participants mostly (70%) had bachelors level qualification with majority (33.3%) having 1–5 years of working experience (Table 1).

**Table 1:** Characteristics of DOs and DMs.

Characteristics		DOs (n = 30)	DMs (n = 17)
		Frequency (%)	Frequency (%)
Gender	Male	10 (33.3)	10 (58.8)
	Female	20 (66.7)	7 (41.2)
Age group (years)	20–30	15 (50)	4 (23.5)
	31–40	9 (30)	8 (47.1)
	41–50	3 (10)	3 (17.7)
	51–60	2 (6.7)	2 (11.7)
	above 60 years	1 (3.3)	0 (0)
Ethnicity	I-taukei	3 (10)	8 (47.1)
	Fijian of Indian Descent	24 (80)	8 (47.1)
	Others	3 (10)	1 (5.8)
Highest qualification	Bachelor level	21 (70)	16 (94.1)
	Post graduate level	9 (30)	1 (5.8)
Number of Years of Practice	6 months—1 years	2 (6.7)	0 (0)
	1–5 years	10 (33.3)	3 (17.7)
	6–10 years	7 (23.3)	3 (17.7)
	11–20 years	7 (23.3)	10 (58.8)
	21–30 years	3 (10)	1 (5.8)
	More than 30 years	1 (3.3)	0 (0)

There were more male participants (DMs) (58.8%) compared to female participants (41.2%). Majority participants were from the age range of 31–40 years (47.1%) while only 11.7% fell in the age range of 51–60 years. 47.1% participants were Fijians of Indian Descent, 47.1% were I-taukei while 5.8% belonged to other ethnicities. Participants mostly (94.1%) had bachelors level qualification with majority (58.8%) having 11–20 years of working experience (Table 1).

### Themes identified for DOs or DMs

Seven themes emerged from data analysis: range of services delivered, appointment versus walk-in patients for aerosol generating procedures (AGPs), impact of pandemic on clinic opening hours, impact of COVID-19 on patient numbers, quality of services delivered, resources and infrastructure, perceptions about the burden of disease. Table 2 summarizes the themes and codes.

**Table 2:** Themes and codes identified for DOs and DMs.

Themes	Open codes	Excerpts examples
**Range of services delivered**	Emergency services, Pain relief, Aerosol generation, Aerosolized procedures, non-Aerosolized procedures, AGPs, ART, Levels, No community transmission, First wave, Second wave, Guidelines, Regulations	*“So*, *in terms of service during this second wave……*.*”* *“Yes*, *there was a time when Fiji became covid free……”* *“Because we are on level 3*, *its emergency treatments…*.*”* *“Aaaa…*. *at the moment we are at level…”* *“…*.*now when there are cases…*..*”* *“During the second wave…*.*”* *“At the moment we are offering just the emergency relief of pain…*.*”* *“In terms of the range of services the…”*
**Appointment versus Walk-in patients for aerosol generating procedures (AGPs)**	Aerosolized procedures, Non- Aerosolized procedures, Cleaning, filling, AGPs, ART, Appointment basis	*“Its 90% appointment basis*. *Most of the time…*.*”* *“Coming towards the beginning of January…”* *“For restorations…*. *because we missed out for all*..*”* *“We mostly see appointment cases…”*
**Impact of pandemic on clinic opening hours**	First wave, second wave, Clinic was closed, clinic was open, Normal operating hours, re-opening of clinics.	*“So*, *during the first wave that was 2020…*.*”* *“Aaaa after the first wave we restricted to only non-aerosol…*.*”* “*The clinic re-opened in August and was…*.*”* *“I think especially for us as a student…*.*”* *“The practice was only close for 3 weeks…*.*”* *“When we got hit by the second wave*, *we…”* *“And then in June*, *we continued…”* *“What we did was*, *before we were working from 8–5…*..*”*
**Impact of COVID-19 on patient numbers**	Reduced patient numbers, decrease in patient numbers, influx of patients, first wave, second wave.	*“So*, *I would say in terms of student’s requirements…”* *“We were having a lot more patients…*.*”* *“Now that I am seeing*, *because the lock…*.*”* *“Actually*, *for us*, *there was a slight…*..*”* *“I would say at the peak of the…*.*”* *“But in the last couple of weeks……”* *“Before*, *I think the average number of…*.*”* *“We are restricted with what we can…*.*”* *“Situation is getting worse*, *the patient numbers…*.*”*
**Quality of services delivered**	Infection control, Good quality, Better quality, Wasn’t compromised, Massive improvement, Compromise in quality, Still the same, SOP, Guideline, not up to par	*“Because we as a clinical supervisor we…*.*”* *I think the quality of services was maintained…*..*”* *“In terms of quality*, *the quality of care that…*..*”* *“I would say it was below par…*.*”* *“In terms of quality I think there…*.*”* *“But now the quality of treatment we a…*.*”* *“In terms of quality*, *the quality…*.*”* *“So basically*, *the services that were offered…*.*”* *“Aaaa…*. *To some level there was compromise of quality…*.*”* *“Service needs from the public I would…*.*”*
**Resources and infrastructure**	Resources, Upgrading, Future, Aerosolized procedures, Negative pressure rooms, Rubber dam, High volume suction, well equipped, improvisation of resources and equipment’s	*“And we had also patients…*..*”* *“Rubber dams*, *we didn’t use rubber…”* *“So*, *these*, *rubber dam and high-volume suction…*.*”* *“For rubber dam*, *in ministry setting…*..*”* *“TDC is well equipped with…*..*”* *“We weren’t given provision…*.*”* *“I requested for those special containers…*.*”* *“And for the technicians as well*, *we made…*.*”*
**Perceptions about the burden of disease**	Restricted services, Quality of life affected, Deteriorating, more disease burden	*“We are still not able to do much conservative…*.*”* *“So*, *if patients want conservative…*..*”* *“Let’s say if a patient comes…*..*”* *“So*, *the quality of oral health in the population…*.*”* *“But like 14 days is a lot of …*. *it’s a very…*.*”* *“What I believe*, *I think as much as we……”*

Each participant was given a unique identification number ranging from DO01 to DO30, for DOs and DM01 to DM17, for DMs. The gender and ethnicity of the participants is also indicated in the quotation references. The perception of DOs is discussed first followed by DMs under each theme.

### Theme 1: Range of services delivered

#### DOs perceptions

While some DOs from private dental clinics are providing full range of services, majority DOs from the private dental clinics were performing reduced range of procedures.

*“So*, *in terms of service during this second wave*, *we have also reduced the number of procedures*, *again we are just doing emergency treatment*. *Aaaaaaa… however*, *we have made allowances for emergency root canal as well*. *Probably just sedative ay*… *without the use of handpieces*. *Because I think the main point was to save the tooth and also provide pain relief or alleviate the pain ay…”* (DO4, a 36-year-old, IT)

A few months after the first wave of the pandemic (2020), the DOs of the government dental clinics started delivering aerosolized procedures as well.

*“Yes*, *there was a time when Fiji became covid free*, *and then we got thumbs up from our divisional bosses that we can start aerosolized procedures*, *so then we started*. *That was around 2 months after the first wave*.*”* (DO25, a 26-year-old, FID).

The government dental clinic continued providing emergency services only, during the second wave of the pandemic with added treatments like Atraumatic Restorative Technique (ART) and temporary dressing without the use of handpieces.

*“Because we are on level 3*, *its emergency treatments only*. *Still no aerosolized treatments are provided*, *only ART*, *if ART can be given then they will excavate and place the temporary filling ay*.*…”* (DO29, a 26-year-old, others)

#### DMs perceptions

All DMs of the government dental clinics stated that the clinic was strictly providing emergency dental treatments during the pandemic, during first wave (2020) and second wave (2021).

*“Aaaa…*. *at the moment we are at level 3 with our national oral health guidance document with the COVID 19 pandemic*. *So*, *with level 3*, *we are just providing urgent and emergency treatments*, *so which is basically urgent extractions that we are providing*.*”* (DM16, a 36-year-old, FID)

Most DMs of government dental clinics also reported of doing temporary dressing for the patients after excavating with hand instrument.

*“…*.*now when there are cases*, *we went up to level 3 ay*, *so currently during this pandemic*, *we are not doing any AGPs*, *we are just doing emergency extractions and dressings*, *temporary dressings and ART ay…for other treatments*, *currently they are on hold*, *especially AGPs…*. *oral surgery cases like wisdom tooth removal and all*, *so that is all on hold now ay…*.*”* (DM14, a 40-year-old, IT)

Majority DMs stated that the type of services provided during the second wave of the pandemic (2021) was mainly non-aerosolized, as per directives from the relevant authorities.

*“During the second wave*, *the FDA advised us that we cannot do any fillings*, *just to do extractions*. *During the second wave I am only doing extractions and oral examinations with little bit on prosthetic cases*.*”* (DM7, a 58-year-old, IT)

A few from the private sector highlighted on the use of extra oral radiographs mainly for diagnosis purpose.

*“We have both x-rays*, *intra oral and extra oral so*, *right now because of the situation we are only doing extra oral x-rays*, *so basically*, *we have a panoramic view*, *we have an x-ray machine that does extra oral bitewings*. *Aaaa yeah so basically*, *we are just doing extra oral*, *we are not doing any intra-oral x-rays*.*”* (DM9, a 34-year-old, FID)

A few DMs also reported of performing endodontic stabilization during the pandemic, apart from extractions.

*“At the moment we are offering just the emergency relief of pain*, *which is dental extraction and sedative dressing or temporary filling*. *And endo stabilization for those who opt to save their teeth ay… just to stabilize*.*”* (DM2, a 50-year-old, IT)

However, one of the DMs of the private dental clinic reported of performing almost all sorts of procedures since the clinic is well equipped.

*“In terms of the range of services the only thing we really not doing now is general routine scaling and cleaning*. *Other than that*, *because we have got the new negative pressure machine and stuff*, *we practically doing everything but of course we are more pre-cautious in terms of you know history*, *triaging well and also like more PPE than normal*.*”* (DM4, a 34-year-old, FID)

### Theme 2: Appointment versus Walk-in patients for aerosol generating procedures (AGPs)

#### DOs perception

Majority DOs from the private dental clinics and school clinics stated that all the cases were seen were mostly on appointment basis while in the government dental clinics, this was only true for Aerosol Generating Procedure (AGPs) after the first wave.

*“Its 90% appointment basis*. *Most of the time if patients do walk in and if there is a chair free*, *then we do see them but most of the time we give the walk-in patients an appointment to be seen*. *And it was always like that*, *even before the pandemic*. *But currently*, *there are no walk-in patients*.*”* (DO1, a 29-year-old, FID)

#### DMs perception

All DMs from the government setting added that AGPs were seen on appointment basis mostly.

*“Coming towards the beginning of January this year*, *we started doing cleaning and filling actually but it was on appointment basis and the way we were doing it like…*. *we try to reduce much exposure to patients*, *so how we did was one patient per day or some appointments are given late afternoon so that all our extraction cases will be completed because we don’t want to expose more people*.*”* (DM13, a 28-year-old, IT)

While a few DMs in the government dental clinic were seeing walk in patients for AGPs after the first wave of the pandemic in 2020.

*“For restorations…*. *because we missed out for all those months not doing aerosolized procedures*, *we treat patients as they walked in*, *if they can pay on the spot*, *we’ll do as many as we can on the same day*. *So that was my advice to the staff ay……*. *So in on day we can allow two officers to do fillings on the spot with one sitting*. *So*, *we try to make up for those lost hours and lost service that we had during the first wave ay…*. *So*, *we make sure*, *when the patient comes in and needs 3 or 4 fillings in one sitting and they can pay for it*, *we do it the same time*.*”* (DM10, a 48-year-old, IT)

Almost all DMs from private dental clinics saw patients on appointment basis, for all dental procedures, during the pandemic, however, a few did see walk in cases as well.

*“We mostly see appointment cases but if whichever patient comes in without booking*, *so we advise them to either take an appointment or if they are in real need then they can sit and wait*. *Sometimes some appointment cases don’t turn up*, *and we also call them in the morning before opening the clinic*, *if they are coming or not*. *So*, *once we know if appointments are confirmed or someone has postponed then we take in those walk-in patients*.*”* (DM8, a 37-year-old, FID)

### Theme 3: Impact of pandemic on clinic opening hours

#### DOs perceptions

DOs from the SDOH stated that they were closed during the first wave (2020) of the pandemic and the school dental clinic resumed with better planning.

*“So*, *during the first wave that was 2020 in march*, *so we closed for about 7 weeks and then when it was covid contained then we planned ourselves in the semester break and then opened in second semester*. *So*, *in the second semester we had planned ourselves really well*.*”* (DO8, a 33-year-old, FID)

While some private clinics and school dental clinics were closed during the pandemic, the government dental clinic was still operational and only delivered non-aerosolized procedures. However, majority DOs delivering these services were not pleased about this.

*“Aaaa after the first wave we restricted to only non-aerosol producing procedures only which just included extraction of emergency cases*. *We didn’t do any restorations or conservative treatment which was a bit unfortunate because some of the cases for which we could have saved the patients teeth later on turned irreversible and needed root canals and extractions*.*”* (DO24, a 26-year-old, FID)

While the government dental clinic continued providing services to the public during the second wave of the pandemic (2021), most private dental clinics were closed for alternating periods before they resumed. DOs of one of the dental clinics provided all range of services to the public as they were fully equipped.

“*The clinic re-opened in August and was closed for about 3 months*. *Everything is pretty much almost back to normal since we have everything set up now*.*”* (DO3, a 46-year-old, others)

Almost all private and government dental clinics are operational, however, the school dental clinic is closed even during the second wave, considering the safety of the students and staff.

*“I think especially for us as a student clinic*, *like we have to take extra precautions because of the nature of work we are dealing with students*. *We had to close down the clinic*, *so even now we are not in any form of service in terms of providing treatment to our patients and also in the student clinics*. *So that is completely on hold*.*”* (DO10, a 46-year-old, others)

#### DMs perceptions

While all government dental clinics had the usual operating hours and days during the pandemic, majority of the DMs of private dental clinics stated that there was a brief period of closure during the first wave in 2020 after which the clinic resumed to providing procedures as normal.

*“The practice was only close for 3 weeks and then we were back to normal again*. *So*, *when there were no cases in the community*, *we went back to normal*. *The confidence that there were no cases in the community was basically based on the information from the government and MoHMS*. *All procedures were being carried out as normal after the 3 weeks of shut down once the clinic opened*.*”* (DM4, a 34-year-old, FID)

Almost all DMs of the private dental clinics stated that the period of closure during the second wave in 2021 was more.

*“When we got hit by the second wave*, *we were actually closed for over a month and a half because our clinic was in the containment zone and we live outside of the containment zone so we couldn’t enter*.*”* (DM5, a 29-year-old, FID)

While other DMs of private dental clinics reported the rise in COVID positive cases affected the clinic operating hours.

*“And then in June*, *we continued for few days and then all of a sudden when it started spiking up to 600*, *700 cases then are topped again for 2 weeks thinking that it might become under control but nothing much was happening so I started going again every consecutive day*.*”* (DM3, a 39-year-old, FID)

A few DMs of the private dental practice reported changes in clinic operating hours and days due to the curfew as well.

*“What we did was*, *before we were working from 8–5*, *uumm…*. *but because of this now we are operating only in the morning session 8–1*. *We are now open from Monday to Friday*, *compared to previously when we were open 6 days a week*. *And like with the curfew still on going*, *so I have to give time to my staff to travel back home*, *and also for cleaning up the clinic after the half days of work*.*”* (DM8, a 37-year-old, FID)

### Theme 4: Impact of COVID-19 on patient numbers

#### DOs perceptions

All DOs from the school dental clinic stated that students had seen reduced number of dental cases due to the pandemic and additional precautionary measures in place after the first wave.

*“So*, *I would say in terms of student’s requirements or the number of cases*, *we re-looked at it and therefore we decreased a few requirements for the students*. *The number of patients*, *the hours had reduced after the first wave of pandemic*.*”* (DO6, a 35-year-old, FID).

#### DMs perceptions

Majority government dental clinics were only allowing restricted number of patients in the clinic.

*“We were having a lot more patients been seen during the first wave*, *actually because of no community transmission but during this second wave*, *we are strictly adhering to our number limitations for the day*, *just to allow enough time to ventilate our room between each patient*. *Yeah*! *So*, *there is a limited number of patients seen during the second wave*.*”* (DM16, a 36-year-old, FID)

One of the DMs from an interior government dental clinic stated that the patient numbers were increasing due to borders and restrictions in place.

*“Now that I am seeing*, *because the lock down is in Nausori and also in the interior of Tavua*, *what I notice is the influx of patients*. *So*, *there is an increase in the patient turn out to the clinic compared to before…*.*”* (DM13, a 28-year-old, IT)

Another DM from a government dental clinic stated that the clinic had seen a rise in patient numbers since they were catering for a large number of populations.

*“Actually*, *for us*, *there was a slight drop during the first few cases… and then it just peaked again*, *like it was really full*. *Like I said before*, *because we serve 3 districts ay…*. *we are always*, *always*, *always full*.*”* (DM12, a 29-year-old, IT)

While most government dental clinics were seeing rise in patient numbers, one of the major hospitals in Fiji, Colonial War Memorial Hospital (CWM), was seeing a decline in patient numbers as the clinic was one of the hotspots during the pandemic.

*“I would say at the peak of the pandemic*, *there was a lesser number of patients presenting to the clinics*. *Basically*, *word got out that our services were limited*, *the government advisories were advising people to stay home and only move only when urgent so that helped as well*.*”* (DM11, a 38-year-old, IT)

DMs of the CWM hospital stated that after seeing decline in patient number for the past 4 months, the patient numbers were increasing slowly.

*“But in the last couple of weeks our patient numbers started to rise again*. *And that’s because it’s been 4 months now*, *any reversible dental condition that anyone would have had at the beginning of the pandemic now would more or less be irreversible so we have seen an increase in the number of patients presenting to the clinics in the last 2 weeks*.*”* (DM11, a 38-year-old, IT)

DMs of the private dental clinics stated that they were limiting the number of patients seen on daily basis during the pandemic.

*“Before*, *I think the average number of patients that we normally see in a day*, *its about 10–15*. *That is even after the first wave*. *Now we have decreased the number of patients*, *we are only seeing about 8 patients in a day*.*”* (DM2, a 50-year-old, IT)

Some DMs from the private dental clinics saw a significant drop in patient numbers due to the limited services being provided during the pandemic.

*“We are restricted with what we can do and…*.*aaa…*. *the patient turnout is also very less so mostly sticking with only emergency treatment*. *We are also doing consultation and advise*, *some prosthetics too*.*”* (DM8, a 37-year-old, FID)

With the significant drop in patient numbers, DMs of private dental clinic expressed their disappointment.

*“Situation is getting worse*, *the patient numbers are decreasing*, *like before our phones would start ringing like 7am in the morning just to get an appointment*. *But now we don’t really get a lot of calls*. *Things are not looking too bright right now*.*”* (DM6, a 29-year-old, FID)

### Theme 5: Quality of services delivered

#### DOs perceptions

All the DOs from the school dental clinics stated that the quality of the services was maintained and wasn’t compromised after they resumed to providing services after the first wave of the pandemic.

*“Because we as a clinical supervisor we are always there supervising with the students so we have not compromised the quality of service to the public in any way*.*”* (DO6, a 35-year-old, FID)

DOs from the school dental clinic felt the quality of services was better due to improved infection control.

*“I think the quality of services was maintained as before the pandemic*, *uumm*. *actually*, *I think our infection control was really improved because of the nature of the pandemic*. *So*, *I would say it was very organized and the infection control practices were really being adhered to compared to pre-pandemic and I think the students also really understood the purposes of the infection control procedures that we have*, *so I think in that sense I think it was good*.*”* (DO9, a 39-year-old, FID)

Majority DOs from the private dental clinics also felt that their quality of services had improved.

*“In terms of quality*, *the quality of care that we provide is definitely the same or even better now because we more cautious*.*”* (DO3, a 46-year-old, others)

While majority DOs from the government dental clinics stated the quality of services had improved, a few the DOs stated that it was below par due to reduced range of services being delivered.

*“I would say it was below par*. *There were so many teeth which we could save but because we were not allowed to use the hand piece*, *we were just extracting teeth*.*”* (DO18, a 26-year-old, FID)

#### DMs perceptions

Majority DMs from the government dental setting stated that the quality of services had improved drastically as compared to before the pandemic due to better infection control procedures being in place.

*“In terms of quality I think there was a massive improvement because considering the infection control that was being practiced during that first wave and then we continued to like work on our infection control bit and then we had our SOP and guidelines in place*. *So*, *I think that the public was served better*.*”* (DM16, a 36-year-old, FID)

Majority DMs of the private dental clinics felt the quality of service was not affected.

*“But now the quality of treatment we are providing now*, *yeah*, *the quality is still the same as in for consultations*, *for extractions*, *and x-rays but we are not providing any other dental treatment*. *The range of treatment basically has been compromised*.*”* (DM9, a 34-year-old, FID)

A few DMs of private dental clinics felt that the quality to some extent was better than before.

*“In terms of quality*, *the quality of care that we provide is definitely the same or even better now because we are more cautious*. *Our quality of service is definitely hasn’t been compromised*.*”* (DM4, a 34-year-old, FID)

A few DMs from the government dental clinics stated that despite the limited information they had and with the limited services offered, they were still able to deliver good quality work to the public.

*“So basically*, *the services that were offered were limited*, *especially those dealing with AGPs aye…but what we could offer was in terms of pain alleviation*, *preventive dentistry*. *Aaaaaaaa quality was good*, *we had to implement a lot of new protocols that were new to us but that was done very effectively with the available information we had and we were still able to treat patient with a good quality of care ay*… . *whether it be just for extraction or temporary filling*.*”* (DM11, a 37-year-old, IT)

While there were some DMs from the government dental clinic expressing their disappointment and stated the quality was compromised to some extent, after the first wave of the pandemic when the clinic had resumed to providing all treatments.

*“Aaaa…*. *To some level there was compromise of quality*, *especially with the provision of conservative treatment*. *We actually were giving appointments for conservative treatments*. *Before COVID every patient who walks in requires conservative treatments*, *we used to provide them then and there but after the first wave of COVID we started giving out appointments*. *So*, *less patients were provided with this service*.*”* (DM17, a 36-year-old, FID)

A few DMs stated that the demand for services from the public continued once the clinic was providing all procedures, once Fiji was COVID contained, but the quality of services was maintained.

*“Service needs from the public I would say was normal but the only thing I would say that was a bit different was the load ay… because of the closure during that period of time*. *But in terms of the quality of services*, *you know we tried*, *we did our best and we still worked in a way that was always practiced from before*, *in terms of like producing quality work*.*”* (DM15, a 53-year-old, IT)

### Theme 6: Resources and infrastructure

#### DOs perceptions

Majority DOs from the government dental clinics stated that they were unable to deliver desirable treatment to patients due to lack of resources. These patients are not able to afford treatments in private dental clinics either, hence, they end up losing their teeth.

*“And we had also patients which could not afford restorations in private clinics that requested for extractions as well and when you deny them*, *after a few weeks you will see them again and the disease by then progresses into the pulp and then we have to take out those teeth as well*. *So*, *in light of the situation*, *it was a bit disappointing that we didn’t have any negative pressure room and the only treatment we were able to offer was extractions*.*”* (DO24, a 26-year-old, FID)

DOs constantly highlighted on the lack of resources to perform conservative treatments with minimal aerosols. DOs in the government dental clinics were asked to improvise rather.

*“Rubber dams*, *we didn’t use rubber dams*. *We didn’t have all the resources*, *we have the rubber dam itself*, *but we didn’t have materials to place the rubber dams*, *but we were told we could use the gloves (laughs)*.*”* (DO29, a 26-year-old, others)

Others thought that lack of resources was putting the practitioners at risk.

*“So*, *these*, *rubber dam and high-volume suction*, *they all compromise our services as there is a high risk of aerosols being generated*, *so high risk of the operator getting infected with the COVID virus*. *Because we don’t have all these services*.*”* (DO16, a 29-year-old, FID)

A few DOs thought the lack of resources to the clinics depended on the supplies.

*“For rubber dam*, *in ministry setting we haven’t been practicing the use of rubber dam*, *so I think we may start but hat all depends on the supplies as well*. *Right now*, *whatever FPBS gives us is what they have when we order*, *if they don’t have those then we make do with whatever we have*.*” (*DO16, a 29-year-old, FID)

While majority dental clinics had issues with resources and infrastructure, one of the private dental clinics was fully equipped along with the school dental clinic. The school has two teaching clinics; TDC (Tuisuva Dental Clinics) and JBS (J.B.Savou).

*“TDC is well equipped with that but our other dental clinic*, *JB Savou*, *did not have that advanced centralized air conditioning unit*, *and that is quite an old clinic as well*, *so that 12 chair clinics did not open for the rest of the year because it did not have that particular ventilation system in place*.*”* (DO8, a 33-year-old, FID)

#### DMs perceptions

Majority DMs from the government dental clinic stated that the clinic was not provided with essentials resources after the first wave (2020) when they had resumed to providing AGPs. However, necessities are being provided currently during the second wave (2021) of the pandemic.

*“We weren’t given provision to things like rubber dam*, *high volume suction or face shield after the first wave*. *But currently we have been given face shield*, *masks*, *and a lot things to assist*, *like hand sanitizers ay*, *and a lot of things to assist and improve our infection control protocols*. *As compared to before*, *we were not so furnished*.*”* (DM12, a 29-year-old, IT)

In terms of resources, one of the DMs from the government dental clinic stated that the clinic had to improvise and create their own resources to minimize the aerosols escaping everywhere. The following innovative ways were undertaken by the clinic manager and team:

*“I requested for those special containers that we use for trimming*, *through the purchasing unit of our government supplies*, *government pharmacy where we get all our stuffs and the biomed unit of the CWM but we couldn’t get any*, *so we custom made one through like an old incubator*.*”* (DM15, a 53-year-old, IT)

Other innovative methods undertaken were:

*“And for the technicians as well*, *we made guards*, *guards so that they are able to trim inside*, *putting the hands from the sides so that the aerosol thing is contained within*. *And then vacuum cleaners as well*. *The suction effect of the vacuum cleaners*, *we got them mounted underneath the working bench*. *That pulls that dust and other things as well ay…”* (DM15, a 53-year-old, IT)

### Theme 7: Perceptions about the burden of disease

#### DOs perceptions

A few DOs from the government dental clinics stated that there are advanced diseases seen due to not being able to deliver appropriate services to the public.

*“We are still not able to do much conservative treatment and as a result we are seeing more cases of advanced odontogenic infections*, *facial cellulitis patients*, *at a rate we had not seen before*.*”* (DO24, a 26-year-old, FID)

Mostly the DOs from the government dental clinic were not so happy with the range of services being delivered, as there was not much the public could choose from.

*“So*, *if patients want conservative treatment your only option is to go to a private dentist*, *but assuming you don’t have the necessary finance*, *then what is your option*? *So*, *the only way I see it is that we have restricted our services to an extent that has made largely the population handicapped to choosing only extractions as the means for pain relief or any dental pathology for that matter*.*” (*DO18, a 26-year-old, FID)

Most DOs from the government dental clinics stated the type of services delivered currently will have implications in future.

*“Let’s say if a patient comes in with a fractured mandible*, *you treat them with closed reduction ok*, *then after you remove the wires*, *they still have malocclusion*, *so definitely their quality of life will be affected*.*”* (DO27, a 34-year-old, IT)

DOs stated that the disease burden in the population will increase.

*“So*, *the quality of oral health in the population might be deteriorating because routine services are not provided such as cleaning or filling*. *So those things are not being provided and that might increase the disease burden in the community*.*”* (DO8, a 33-year-old, FID)

While other participants stated that the pandemic is affecting the people’s quality of life. One of the DOs shared their experiences of seeing a patient who tested COVID positive and was not able to receive treated until the patient was cleared.

*“But like 14 days is a lot of …*. *it’s a very long time for the patient*. *because that time when we initially saw him*, *we didn’t really do an examination but by the looks of it*, *it got aggressive*, *so in these 14 days*, *we have seen that a lot of his tongue structures had got lost ay*, *so if it was before this covid was hit*, *so by now we would have been able to do his operation and you know his quality of life would have been much better*.*”* (DO21, a 29-year-old, FID)

#### DMs perceptions

A few DMs of the government dental clinic felt they were helpless and empathized with the patients as they believed the range of services delivered to the public during the pandemic was not up to par.

*“What I believe*, *I think as much as we are trying to safeguard ourselves from getting exposed*, *uumm…*.. *in terms of what we deliver right now*, *its not up to par with what we have learnt*. *We are just doing extraction ay…*.*so we know like if the cases comes in during the pre-pandemic times*, *instead of just pulling it out*, *we can do root canal*, *we can fill the tooth*, *extraction is like the least option ay…*.*But for now since we are not allowed to do all those…*.*all those treatments that will contribute to the increase of aerosols*, *we have no other choice but we just have to tell the patient that this is the protocol and we are trying to protect ourselves from the exposure*, *so the only option is either extraction or medications*.*”* (DM13, a 28 year old, IT)

## Discussion

This study aimed to explore the perspective of Dental Officers (DOs) and Dental Managers (DMs) on the effects of COVID-19 on dental service delivery in Fiji Islands. Thirty DOs and seventeen DMs were interviewed. The major themes that emerged were: range of services delivered, appointment versus walk-in patients for aerosol generating procedures (AGPs), impact of pandemic on clinic opening hours, impact of COVID-19 on patient numbers, quality of services delivered, resources and infrastructure, perceptions about the burden of disease.

With regards to the range of dental treatments, majority dental clinics provided emergency services, while a few clinics in the private settings were providing limited aerosolized procedures, with extra precautionary measures. Similar finding was reported in literature where results showed that majority dental clinics and hospitals had suspended non-emergency dental treatment and only emergency dental services were provided [4,30–32]. Emergency dental treatment can be performed in the teaching hospital or local public dental clinic in all countries [32]. As for this study, the teaching clinic of SDOH was closed during both the waves of the pandemic and did not provide any patient care during those periods. However, 98% guidance documents, as reported by Robertson et al., (2021) [31] recommended that AGPs can be performed with patients without COVID-19 with caveats, including advice to restrict AGPs where possible. Literature showed that dentists from the private sector saw lesser emergency treatments than those in public sector [33]. This finding was in contrary to the findings from this study. Majority dental treatments were related to pulpal issues, abscess, periapical lesions, cellulitis and trauma [34]. The dental problems were mostly solved through 3A approach (advice, analgesics and antibiotics) and dental extractions [34]. Similar types of cases and approach was taken by majority DOs and DMs in this study. Literatures showed that there was an overall decrease in dental procedure being provided [35,36]. Dental mangers in this study also reported a decrease in majority dental treatments, as per their clinical reports, while there was an increase in dental extractions noted. While majority dental clinics were only providing emergency care, dental clinics in the private dental setting which were fully equipped operated as per the guidance document from Fiji Dental Association (FDA) and provided all range of services, in this study. Similar finding was reported by Schlenz et al., (2021) [37] where one of the universities in Germany continued providing the usual dental care to all patients with increased safety measures. This did not coincide with the dental university in this study, as the School of Dentistry and Oral Health in Suva Fiji was closed during both the waves of the pandemic considering safety of staff and students.

Majority private dental clinics and the school teaching clinic were mostly seeing patients on appointment basis only. However, most government dental clinics were seeing walk in patients as well and were mostly giving appointments for aerosolized procedures only. Majority private dental practices, including the school dental clinic, in this study were closed during the lock down period while the government dental clinics were still operational as part of essential services. Similar finding was presented by Tada et al., (2021) [3], whereby the dental clinics were not closed and continued providing treatment which was a little different from usual. Similarly, the dental settings in this were providing limited services. Chang et al., (2021) [32] reported the closure of most private dental clinic in most of the countries, except in Taiwan. A lot of private dental clinics in this study reported of reduced operational hours and reduced number of days they were open. Similar finding was reported by Al Kawas et al., (2020), whereby 84.2% dentists reduced their hours of services [38]. There was no change in the operation hours for the government dental clinics in this study, however, a few DMs did give an early break to their staff as they worked through lunch hours to limit wastage of PPEs.

Majority DMs and DOs experienced lesser number of patients in the dental clinic during the peak time of the pandemic. Patient numbers began to rise slowly. However, the patient numbers differed in different clinics according to geographical locations. This was due to border restrictions put in place by the government. Similarly, other studies also showed a decline in patient numbers [33,39]. Wolf et al., (2021) [35] stated that private dentists had fewer emergency patients per week compared to public health dentists. However, the contrary was noted in this study whereby more patients were seen in the government dental clinics than private. Moraes et al., (2020) [40] stated the reduction in the number of patients was greater in public (38.7±18.6) than in private clinics (22.5±17.8). Prevention protocols were put in place which enabled the number of patients return to the normal range with a slight increase during the post pandemic period. There wasn’t any documented case of COVID-19 related to dental treatment in the hospital [40]. None of the DOs and DMs reported of any incidents of COVID-19 from the dental clinics in this study as well. While majority dental clinics were seeing a reduction in patients’ numbers, approximately 50% participants in a study did not see any decrease in in visits since the outbreak spread [41].

Majority DOs and DMs in this study agree that their quality of emergency services had improved due to improvements in infection control. A few others stated that the level of care remained the same. While a few participants were worried about the range of services being provided, hence, stated that the quality of services in terms of range can improve. Schlenz et al., (2021) [37] reported of that the patient care was maintained and no changes were noted in terms of the quality of care. However, Wolf et al., (2021) [35] stated that the level of care was impaired.

Lack of resources and infrastructure became a hindrance for the provision of dental services in the government dental clinics. The DOs in the government dental clinics had to improvise and adjust with the available resources as they were not provided with the needed resources to safeguard them during the pandemic. While the government dental clinics and majority private dental clinics were facing difficulty with the resources and infrastructure, the school dental clinic and one of the private dental clinics was well equipped and had the necessary resources to proceed with the dental treatments during the pandemic. A few participants in this study were generally worried about the increasing disease burden and the patient’s quality of life since only limited dental services were being provided. Although there were no direct studies done on the quality of life of patients receiving dental treatment during the pandemic, studies reported that participants did not complain about any treatments or suspension of services during the pandemic [14,37].

### Limitations

The in-depth interviews were conducted via zoom instead of face-to-face interviews, due to the pandemic. The study was limited to central division only, hence, perspectives of DMs and DOs outside this division hasn’t been included.

## Conclusion

Dental clinics and service delivery were adversely affected due to the pandemic. Majority dental clinics, excluding the government dental clinics, were closed during the pandemic. It took weeks to months for the practices to open again. While the MoHMS dental clinics were operational, only non-aerosolized procedures were being delivered during both the waves. Most private and school dental clinics were seeing patients on appointment basis. Majority participants saw a drop in the patient numbers during the peak days of the pandemic. DMs also made an effort to only allow limited patients per day so that the dental staff could strictly adhere to the additional protocols in place. Majority participants felt their quality of services had improved, a few others felt that quality can be improved, in regards to the range of services being delivered to the public, as this, they felt is greatly affecting the quality of life of the patients and will have consequences in the long run.

This was a qualitative study done to explore perspective of DOs and DMs. A quantitative study can be conducted to record actual findings. Furthermore, the study was only conducted in central division of Fiji Islands. Future research can be conducted in other divisions and include other health care professionals as well apart from just DOs and DMs.
